# Experimental snowball earth viscosity drives the evolution of motile multicellularity

**DOI:** 10.1016/j.isci.2026.117756

**Published:** 2026-10-08

**Authors:** Andrea Halling, Brysyn Goodson, Anna Hirschmann, Boswell A. Wing, Carl Simpson

**Affiliations:** 1 Science Research Initiative, University of Utah, Salt Lake City, UT 84112, USA; 2 Department of Integrative Biology, Oregon State University, Corvallis, OR 9733, USA; 3 Department of Ecology and Evolutionary Biology, University of Colorado, Boulder, CO 80309, USA; 4 Department of Applied Mathematics, University of Colorado, Boulder, CO 80309, USA; 5 Department of Earth Science, University of Colorado, Boulder, CO 80309, USA; 6 Museum of Natural History, University of Colorado, Boulder, CO 80309, USA

**Keywords:** snowball earth, multicellularity, viscosity, eukaryotic flagella, division of labor

## Abstract

During the 70-million-year Cryogenian Snowball Earth glaciations, low ocean temperatures beneath global sea ice increased water viscosity over 3-fold. Unicellular eukaryotes in this environment would have faced severe constraints on movement and nutrient uptake, while nascent multicellular organisms, with larger size and collective mobility, could have gained an adaptive advantage. We experimentally tested whether viscosity-induced metabolic constraints could have selected for multicellular phenotypes, potentially driving the origin or early diversification of multicellularity. Under Snowball Earth-like viscosities, populations of unicellular green algae evolved motile multicellular forms, along with other phenotypes that optimize size and speed. As oceans warmed and viscosities decreased, the diversity of multicellular phenotypes may have allowed early multicellular organisms to exert novel physical control over their local environments. This pivotal period in Earth’s history could have shaped the emergence and eventual dominance of certain multicellular lineages, highlighting a potential ecological driver of one of life’s major evolutionary transitions.

## Highlights

• Multicellularity evolves across a range of Snowball Earth viscosities
• Multicellular forms retain the motility of their unicellular ancestor
• Multicellular life cycles are observed to be due to failed separation after mitosis

## Graphical abstract

**Figure:**
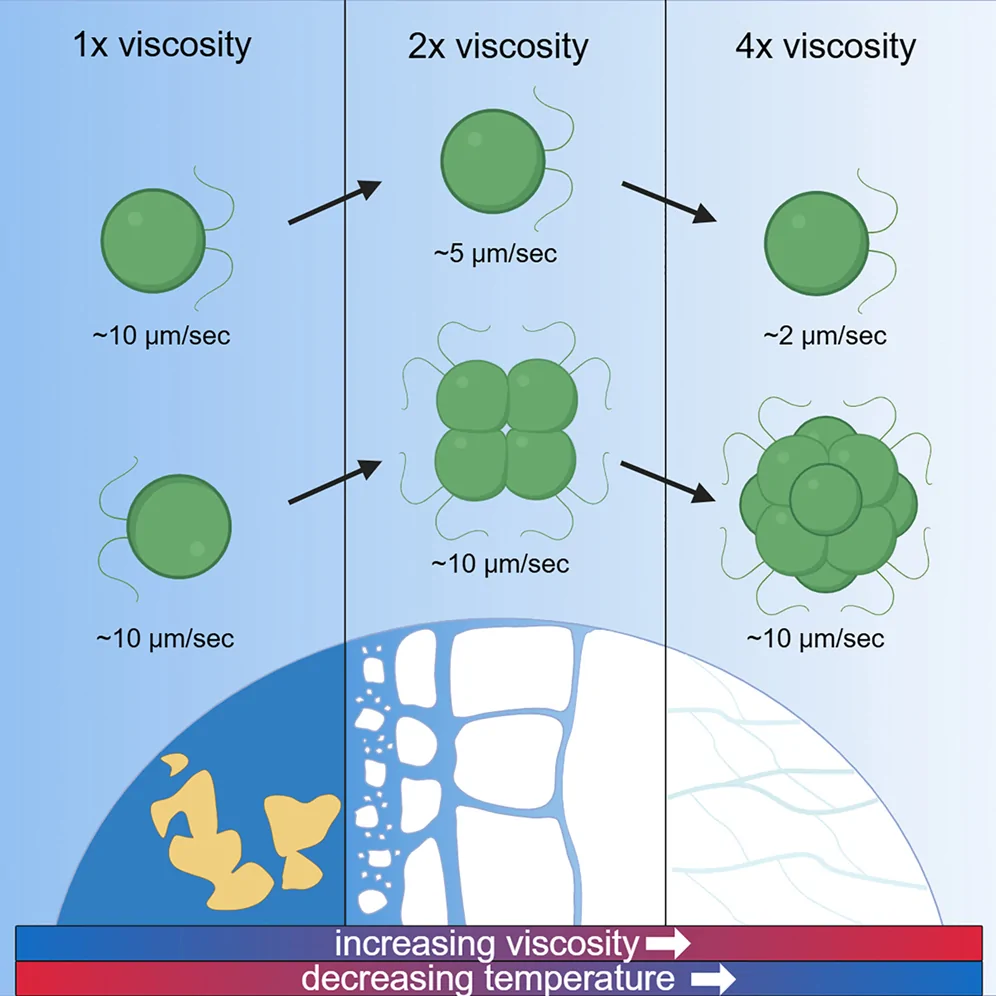

## Teaser

Earth sciences; Geology; Paleontology; Global change; Earth history; Glaciation in earth history; Paleobiology

## Introduction

Multicellular life in its most simple definition (i.e., having multiple cells) has evolved many times in Earth’s history^1^^,^^2^ and easily evolves in response to numerous experimental selective pressures in the lab.^3^^,^^4^^,^^5^^,^^6^^,^^7^^,^^8^ However, out of well over 25 independent multicellular origins in eukaryotes, only a few have become ecologically dominant.^9^^,^^10^^,^^11^^,^^12^ This highlights a fundamental evolutionary question: if multicellularity is so easy to evolve, why did the modern complex lineages that have dominated ecosystems over the last 540 million years take so long to diversify after the origin of eukaryotes? Investigating the temporally unique geological selective pressures when modern plant and animal lineages first began to radiate can help to resolve this question.

### The Tonian-Cryogenian radiation of plant and animal clades

All sources of information on the origins of plant and animal lineages are biased in different ways, but the direction of those biases helps paint a clearer picture.^13^ Molecular clock dates—which tend to be biased toward older dates^14^^,^^15^^,^^16^^,^^17^—indicate that the origins of animals occurred within the Tonian, prior to the Snowball Earth Glaciations.^18^^,^^19^ Early fossilized animals—which tend to be biased toward more recent dates^15^^,^^20^^,^^21^—appear in the Cryogenian interglacial interval^22^ as well as the Ediacaran after the Snowball Earth glaciations are over.^23^^,^^24^

The molecular clock dates of multicellular crown-group green and red algae are clustered in the Tonian-Cryogenian.^25^^,^^26^ Although many fossils of eukaryotic algae with coenocytic or multicellular organization appear earlier in the rock record,^1^^,^^27^^,^^28^^,^^29^^,^^30^^,^^31^^,^^32^^,^^33^ they remain relatively small in size and at very low diversity until filamentous multicellular algae increase in abundance and diversity in the Tonian^28^^,^^34^ (Figure 1). In the Cryogenian interglacial Nantou flora, larger algal fossils appear with a significant increase volume.^28^^,^^42^ Uncertainty nevertheless dominates the Proterozoic record of multicellular photosynthetic eukaryotes. This uncertainty arises both from the incomplete nature of the fossil record^43^ and from difficulty distinguishing coenocytic, multinucleate, and multicellular algal forms.^34^^,^^44^^,^^45^

**Figure 1:**
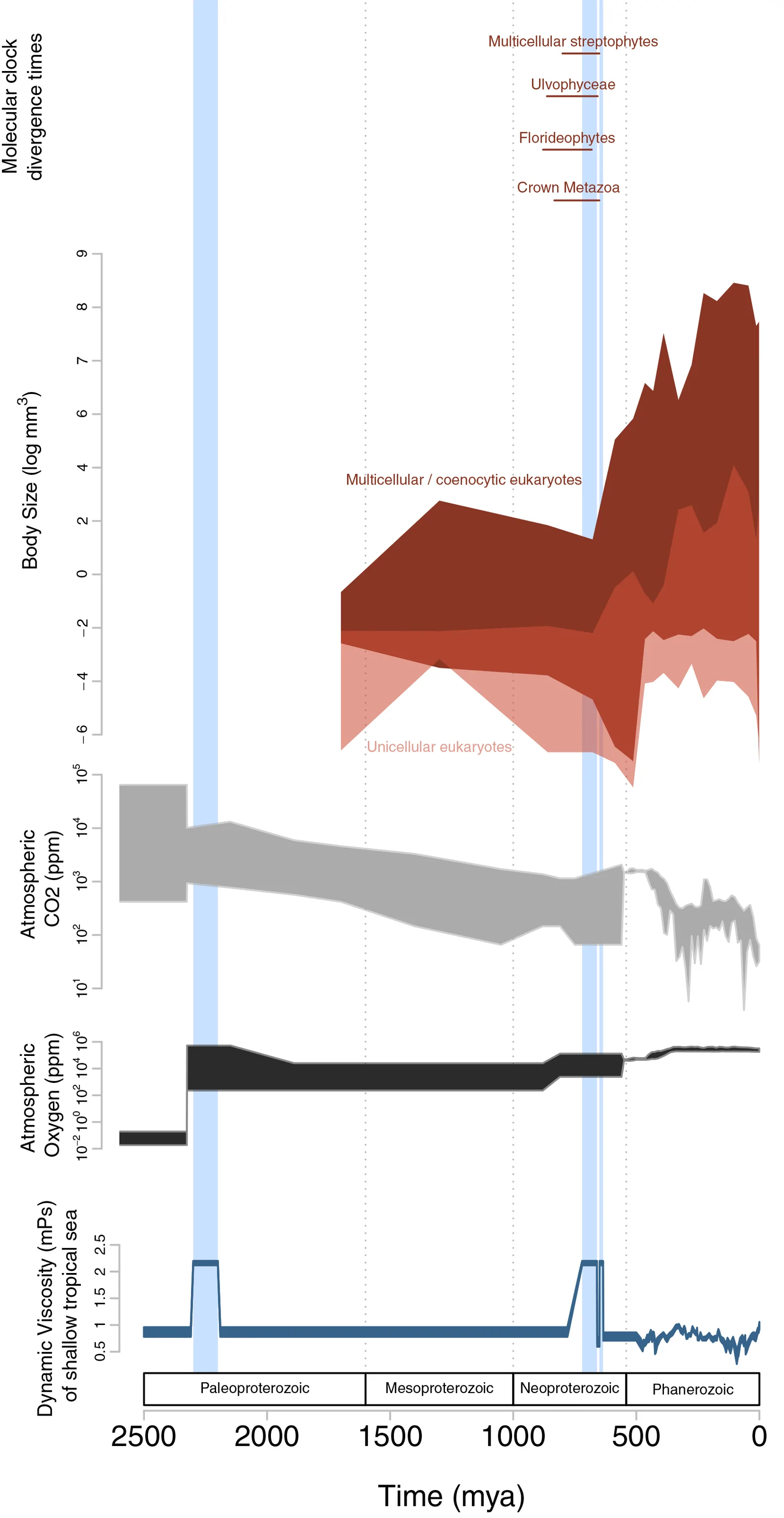
Schematic depicting the evolution of multicellular eukaryotes and contemporaneous changes to the Earth System over the last 2.5 billion years Divergence times of clades possessing complex multicellularity, body sizes of unicellular and multicellular/coenocytic eukaryotes, inferred and measured CO_2_, oxygen, and tropical seawater viscosity levels are shown over Neoproterozoic and Phanerozoic time. We show 95% confidence intervals for molecular clock divergence time estimates for multicellular lineages.^18^^,^^25^^,^^26^ We show maximum and minimum body volume (log mm^3^) of fossils of unicellular and multicellular (or coenocytic) eukaryotic genera observed or inferred to range through an interval of time.^23^^,^^28^^,^^35^^,^^36^ Proxies and modeling provide estimates for maximum and minimum ranges of CO_2_ ppm^37^^,^^38^^,^^39^ and O_2_ ppm.^38^^,^^39^ Inferred seawater viscosity for the shallow equatorial ocean with glacial interval viscosities for seawater with temperatures from −4 to −3 °C and salinities of 55 ppt. Interglacial seawater is shown with 35 ppt seawater and temperatures ranging from 25°C–35 °C.^40^ Latitudinally constrained SST data and models are available for the Phanerozoic^41^ and used to calculate the viscosity of tropical surface water assuming salinity was 35 ppt over the Phanerozoic.

Molecular clock analyses allow for a possible Tonian divergence time for animals; however, no convincing animal fossils have been identified from the Tonian or earlier, despite the presence of abundant and well-preserved sedimentary rocks that should record their existence if they were present.^24^ Although the taxonomic affinities of the earliest candidate animal biomarkers and body fossils remain uncertain, these records appear in the Cryogenian.^22^^,^^46^ These observations suggest that the origin of animals and the major expansion in disparity and diversity among multicellular algae coincide temporally with the Cryogenian Snowball Earth interval (Figure 1).

### Prevailing hypotheses for the origin and evolution of complex multicellularity

The oxygen requirement of modern multicellular organisms, particularly animals, has long led to the inference that oxygen levels increased along with the rise of animals and other multicellular organisms.^2^^,^^47^^,^^48^ We now know that the significant rise of atmospheric oxygen levels occurred after the Cambrian explosion^49^^,^^50^^,^^51^^,^^52^^,^^53^^,^^54^^,^^55^, nearly 200 million years too late for it to be the cause of the origin of multicellularity in animals and 800 million years too late for multicellular algae. In addition to this mismatch in timing, high seawater temperatures in the aftermath of the Snowball Earth glaciations^56^ would have physiologically limited access to oxygen.^57^^,^^58^^,^^59^ Therefore, the ability for animals to use any increasing oxygen levels would have been suppressed by thermal stress until the oceans cooled sufficiently.^60^ Moreover, experimental evolution studies indicate that elevated oxygen levels can actually suppress the growth of multicellular forms.^61^ This counterintuitive result arises from first principles. At very low oxygen, growth is slow but unconstrained because all cells ferment. As oxygen increases, a fraction of cells respire while the remainder ferment, and this metabolic mix imposes stronger constraints on size until levels become high enough to relax this constraint again. Theoretical work also suggests this pattern is likely general rather than idiosyncratic.^61^

It has also been hypothesized that continental weathering caused by the Snowball Earth glaciations resulted in a massive increase in nutrient availability,^62^^,^^63^ favoring the evolution of multicellularity. But as with oxygen, high nutrient levels likely arose much earlier.^64^ Evidence for high phosphate levels is evident in hydroxy-apatite biomineralization in Tonian seawater.^65^^,^^66^^,^^67^ Cryogenian nutrient influx does well to explain the shift from predominantly prokaryotic to eukaryotic primary productivity,^62^^,^^68^ but does not easily explain the origin of complex multicellularity, even if it helps explain the later Cambrian explosion.^69^ Biotic drivers, such as predation and grazing, are also a common hypothesis for the selection of multicellular eukaryotes.^4^^,^^5^^,^^6^^,^^70^ Yet, eukaryotic predation has existed since eukaryogenesis, long predating the origin of eukaryotic multicellularity.^71^^,^^72^^,^^73^ It is also worth noting that unicellular autotrophs often possess the ability to curtail predator-prey arms races^74^ beyond forming colonies, for example by excreting deterrent compounds, or coupling larger size with lower nutrient density so that predation pressure is reduced.

### Multicellularity as an adaptive strategy for snowball earth ocean viscosity

Seawater viscosity is largely determined by temperature, with a smaller contribution of salinity, and can be described by the Vogel-Fulcher-Tammann equation^75^ (Figure 2). The temperature variation in the ocean was substantial, and the time it took the Earth to cool as it entered the Sturtian glaciation is unknown. Nevertheless, it is possible to put some bounds on what could have occurred. While it is possible that the rate of cooling was extremely fast, climate models^77^ and some newer geological evidence suggest a longer more protracted period of cooling.^78^^,^^79^ There is also uncertainty in the extent of glaciation during the Cryogenian. The two prevailing hypotheses are a “soft snowball” could have occurred with a cold and slushy, but ultimately ice-free, equatorial ocean, or a “hard snowball” with a colder subglacial ocean and ice covering the equator. To factor in these various possibilities, we consider the maximum and minimum ranges for the flux in dynamic viscosity. In the most extreme case we have a “hard snowball” scenario with hot temperatures pre-glaciation (45°C) with a salinity of 35 ppt.^56^ Based on model estimates, ocean temperatures may have fallen from over 45 °C to about −5 °C as salinity increased from 35 ppt to at least 45–50 ppt.^40^ At this extreme, viscosity would have increased roughly 4-fold, although it is unlikely that many eukaryotes could survive at 45 °C eukaryotes are known to live up to 65 °C.^80^^,^^81^ In a more moderate “soft Snowball” scenario, where ocean temperatures were already cooling before glaciation,^78^^,^^79^ temperatures may have dropped from around 30 °C–0 °C. Such a change would lead to more than a 2.25-fold increase in viscosity. For comparison, seawater at −4 °C and 45 ppt salinity is about twice as viscous as seawater at 19 °C and 35 ppt.

**Figure 2:**
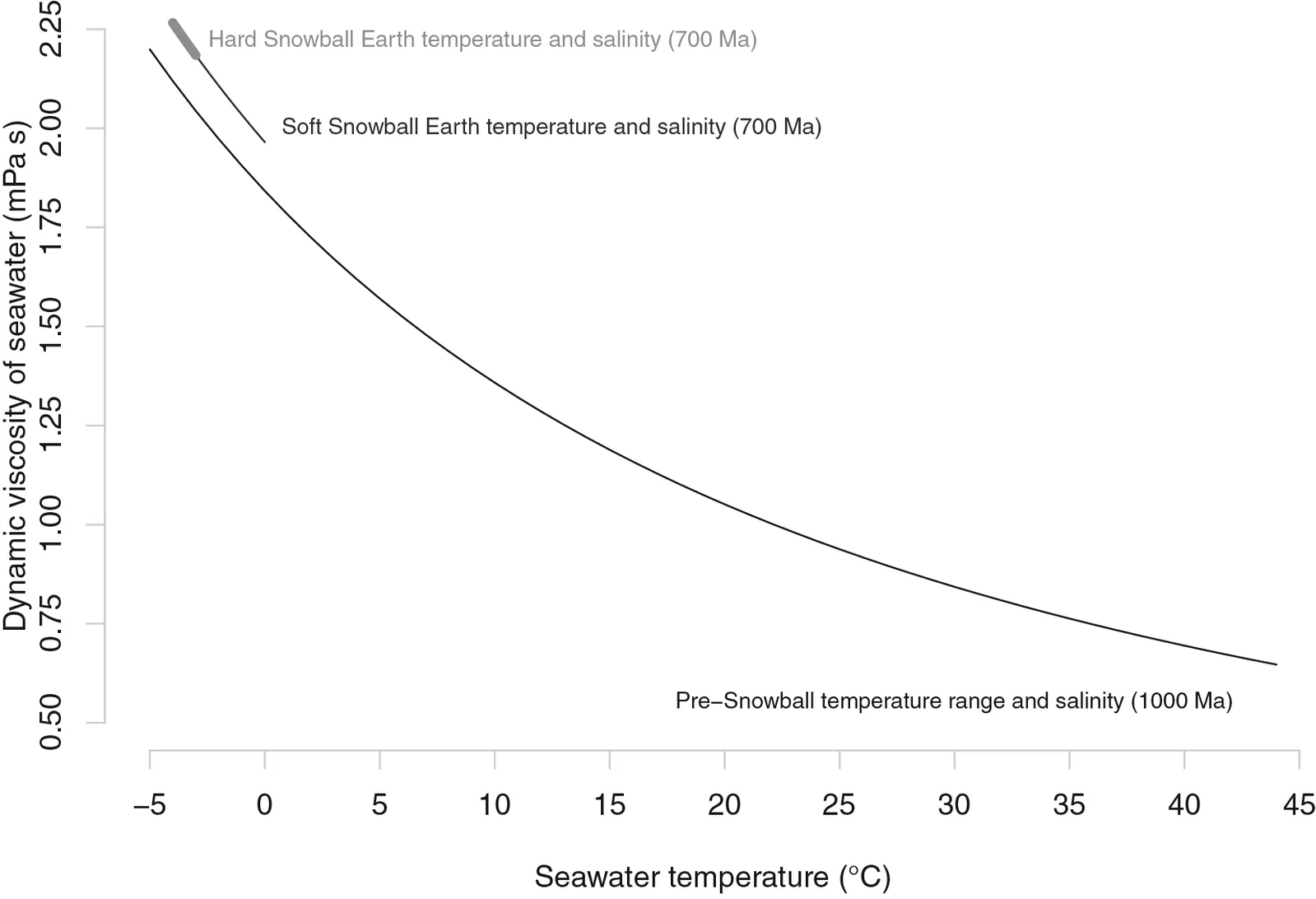
Dynamic viscosity profiles of seawater for preglacial and Snowball Earth oceans Viscosities are based on reconstructed sea-surface temperatures and salinities for preglacial (−4°C through 45°C, 35‰ salinity) and for soft (−4°C through 0°C, 50‰ salinity) and hard (−4°C through −3°C, 50‰ salinity) Snowball oceans. Dynamic viscosity is shown in units of millipascal seconds (mPa s).^76^

While cold seawater surely existed at depth and latitude pre-glaciation, the lack of habitat diversity was unique to the Snowball Earth. Life could survive under thick sea ice, but to access nutrients, organisms would have had to contend with the high viscosity of the seawater. Proposed refugia, including terrestrial and cryoconite-hosted microhabitats,^76^^,^^82^ may have provided localized shelter, with cryoconite pans potentially accumulating ions via atmospheric deposition over the long glaciation. Even so, the vast extent of the ocean makes a marine refugium the more likely setting for eukaryotes. Despite an unknown mechanism for maintaining photosynthesis under thick sea ice, the levels of light needed are lower than typically appreciated.^83^ With little evidence for widespread extinction,^84^ Cryogenian eukaryotes must have adapted to these conditions to overcome the metabolic deficits imposed by diffusion limitation in cold, viscous seawater.

There are several hypothesized strategies that unicellular organisms could have used in order to minimize this deficit by optimizing size, motility, and metabolic scaling.^35^^,^^85^ Unicells could have become smaller, lowering their metabolic rates to match availability. At constant size, maintaining metabolism in a more viscous medium would drastically slow nutrient encounter rates and thus growth; to maintain comparable growth rates, smaller cells with lower total metabolic demand would be favored. Alternatively, unicells could have increased nutrient acquisition by becoming multicellular. Larger sizes together with higher collectively generated movement (either moving to nutrients through motility, or nutrient movement through flow generation) would have increased encounter rates enough to satisfy metabolic needs of larger bodies.^35^ Any pre-existing multicellular morphologies would have also had this same ecological advantage. Because temperature and viscosity covary (Figure 2), one might expect reduced metabolic costs in cold seawater due to temperature-driven slowing of metabolic rates. However, in eukaryotes the effects of increased viscosity outweigh those of temperature,^86^^,^^87^^,^^88^^,^^89^ and we therefore expect a substantial metabolic deficit even after accounting for the metabolic effects of cold.^85^ While there is likely a combination of factors that contributed to the emergence of multicellularity, the evolution and adaptive selection driven by the physical changes of seawater during the Snowball Earth has the potential to help explain why complex multicellularity arose predominantly (though not exclusively) among eukaryotic lineages.^85^

To test the biological feasibility of this viscosity hypothesis for eukaryotic multicellularity, we isolate the physical aspect of viscosity to use as a selective regime in microbial evolution experiments. Our experimental Petri dish (Macroplate, Figure 3, inspired by Baym et al.^90^) utilizes a stepwise viscosity gradient. This Macroplate spatially represents the temporal increase in viscosity as Snowball Earth conditions set in. Our setup provides a stable gradient over multiple weeks as diffusion of our viscosity-inducing polymer (Ficoll400) occurs as a function of length squared. Wildtype *Chlamydomonas reinhardtii*, a unicellular green alga, was used as a model organism representing a general motile eukaryotic phenotype. We made this choice for two key experimental reasons. First, the scaling parameters of the viscosity model^35^ are already known for both *Chlamydomonas* and the multicellular volvocine algae,^91^^,^^92^ allowing for direct comparisons between theory and experiment. Second, because *Chlamydomonas* is primarily autotrophic and only partially reliant on flow for its metabolism,^93^ it offers the most conservative test of our hypothesis. We inoculated *C. reinhardtii* into HSA media of standard viscosity (0.96 cP at 25°C, we refer to this as our 1× relative viscosity) in the center of the circular Macroplate. The second ring of the Macroplate has a dynamic viscosity of 2.3 cP, a roughly 2× increase relative to our starting viscosity. The outer rings have dynamic viscosities of 4.1 cP—a roughly 4× increase in viscosity relative to where we inoculated. As the population grows out radially, only individuals with the ability to move into the sections of higher viscosity continue to propagate. The spatially structured environment of the Macroplate acts as a vehicle for experimental evolution, with viscosity as the selective barrier, and nutrient availability as the evolutionary driver.

**Figure 3:**
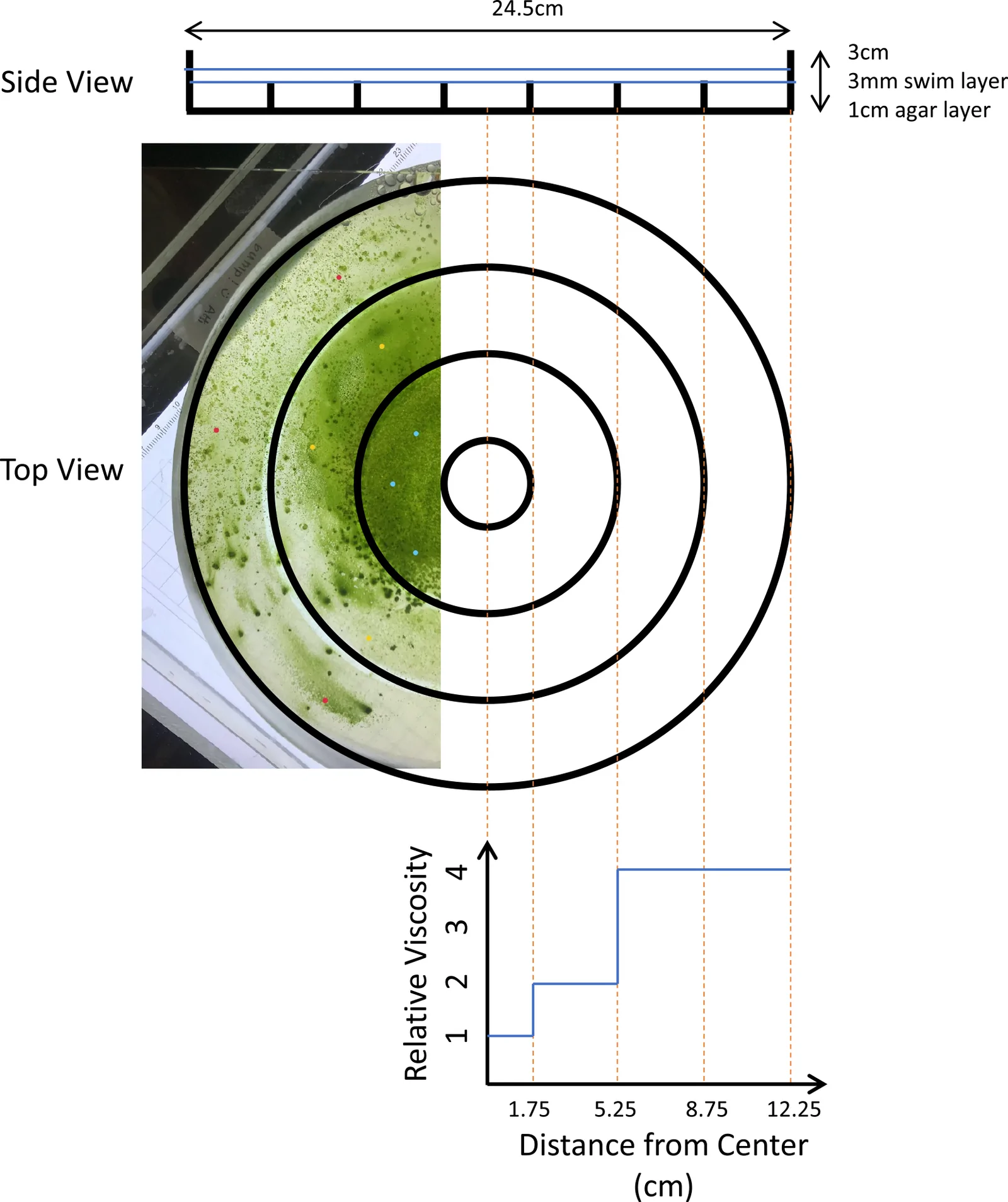
Macro-plate experimental set-up from top and side views The Macroplate, a 24.5 cm diameter Petri dish, featured radial step changes in viscosity (3.5 cm ring width). Ficoll400 in 1.5% agar HSA medium created a solid base layer with varying viscosities. After three days of equilibration, the plate maintained stable sections with 1×, 2×, and 4× viscosities relative to water at 25 °C. *Chlamydomonas reinhardtii* culture (100 μL) was inoculated at the center, and growth over 30 days revealed distinct viscosity gradients, reaching 4× viscosity in the outer ring.

## Results

### De novo evolution of multicellularity in snowball Earth viscosities

We observe the *de novo* evolution of motile multicellular phenotypes in response to a viscosity increase analogous to Cryogenian conditions. This finding provides the first geologically plausible mechanism for the selection of eukaryotic multicellularity.

Under 2× and 4× relative viscosities, *C. reinhardtii* evolves a diversity of phenotypic responses, including flagellated motile colonies of four and eight cells, small non-motile unicells, and macroscopic non-motile colonies. Coulter Counter size distributions as well as microscopy of the populations living within the 2× and 4× sections of the Macroplate reflect this phenotypic shift (Figure 4A). We used three different Coulter Counter aperture tubes (200μm, 1000 μm, and 2000 μm) to capture the range of sizes present in the populations. Large individuals, detectable by the 1000 μm and 2000 μm aperture tubes, were observed exclusively at high viscosities (1000 μm: *χ*^2^(2) = 480.09, *p* < 2.2 × 10^−16^; 2000 μm: *χ*^2^(2) = 92.98, *p* < 2.2 × 10^−16^). Additionally, size distributions measured with the 200 μm aperture tube differed significantly among viscosity treatments (two-sample Kolmogorov-Smirnov tests: 1× vs. 2×, *D* = 0.451, *p* < 2.2 × 10^−16^; 2× vs. 4×, *D* = 0.362, *p* < 2.2 × 10^−16^; 1× vs. 4×, *D* = 0.171, *p* < 2.2 × 10^−16^). In contrast, populations directly inoculated into high-viscosity media did not exhibit the same phenotypic changes (Figure 4B). Comparisons between evolved and plastic populations revealed significant differences in the presence of large individuals for both the 1000 μm and 2000 μm aperture tubes (Fisher’s exact tests: 1000 μm, *p* = 4.998 × 10^−4^; 2000 μm, *p* = 4.998 × 10^−4^). Large colonies were rare in these plastic populations and, when present, were primarily palmelloids; motility was entirely absent.

**Figure 4:**
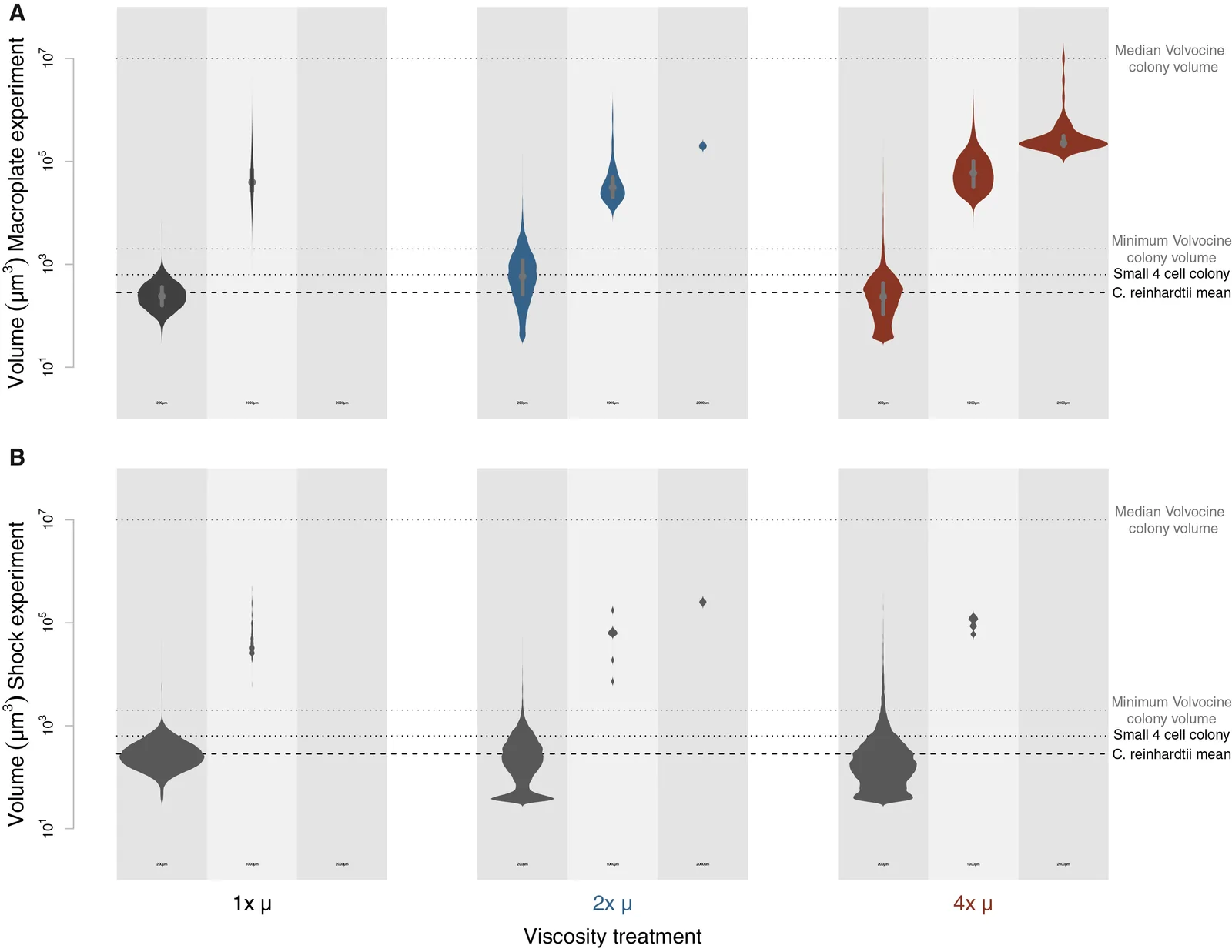
Size distributions of experimentally evolved *C. reinhardtii* and plastic phenotypic responses of *C. reinhardtii* to increases in viscosity of their media (A) Evolution experiments were conducted in a spatially structured Macroplate, 24.5 cm in diameter, with three concentric rings of 1×, 2×, and 4× relative viscosities. The population was inoculated at the center zone with 1× viscosity and grew for 30 days, corresponding to ∼70 generations (10.5-h generation time). Volume measurements from over 13,000 individual organisms sampled from a radial section are shown. Because organisms in this experiment span 6 orders of magnitude in volume, we show separate size distributions for three sizes of Coulter counter aperture tubes—200 μm, 1000 μm, and 2000 μm—in order to cover the range of sizes. Sample sizes for 1× viscosity, 200 μm aperture tube: *n* = 10117; 1000 μm aperture tube: *n* = 0; 2000 μm aperture tube: *n* = 0. Sample sizes for 2× viscosity, 200 μm aperture tube: *n* = 10503; 1000 μm aperture tube: *n* = 298; 2000 μm aperture tube: *n* = 11. Sample sizes for 4× viscosity, 200 μm aperture tube: *n* = 11701; 1000 μm aperture tube: *n* = 698; 2000 μm aperture tube: *n* = 239. For reference, the range and median volume for colonial volvocine algae^94^ are shown as horizontal reference lines. Large individuals, detectable by the 1000 μm and 2000 μm aperature tubes, were observed exclusively at high viscosities (1000 μm: *χ*^2^(2) = 480.09, *p* < 2.2 × 10^−16^; 2000 μm: *χ*^2^(2) = 92.98, *p* < 2.2 × 10^−16^). (B) Phenotypic plastic responses of *Chlamydomonas reinhardtii* populations directly inoculated in environments with differing viscosities (1×, 2×, and 4× relative viscosities). Size distributions, as quantified by Coulter Counter analysis, and microscopic observations reflect the observed phenotypes in response to media with 2× and 4× relative viscosities. Coordinated motility was not observed while typical stress-induced palmalloid phenotypes as well as small, non-motile single cells were present.

### Evolved multicellular organisms exhibit multicellular life cycles

Additionally, under time-lapse microscopy, we observe our evolved motile multicells to have life cycles where individuals produce multicelled offspring (Figure 5). These life cycles are not observed in the palmelloids. Even after 70 generations back in 1× viscosity media, the motile phenotypes persist, while the large, non-motile individuals (presumably stress-induced palmelloids) are lost (Figure 6). This shows that motile multicellular forms are heritable.

**Figure 5:**
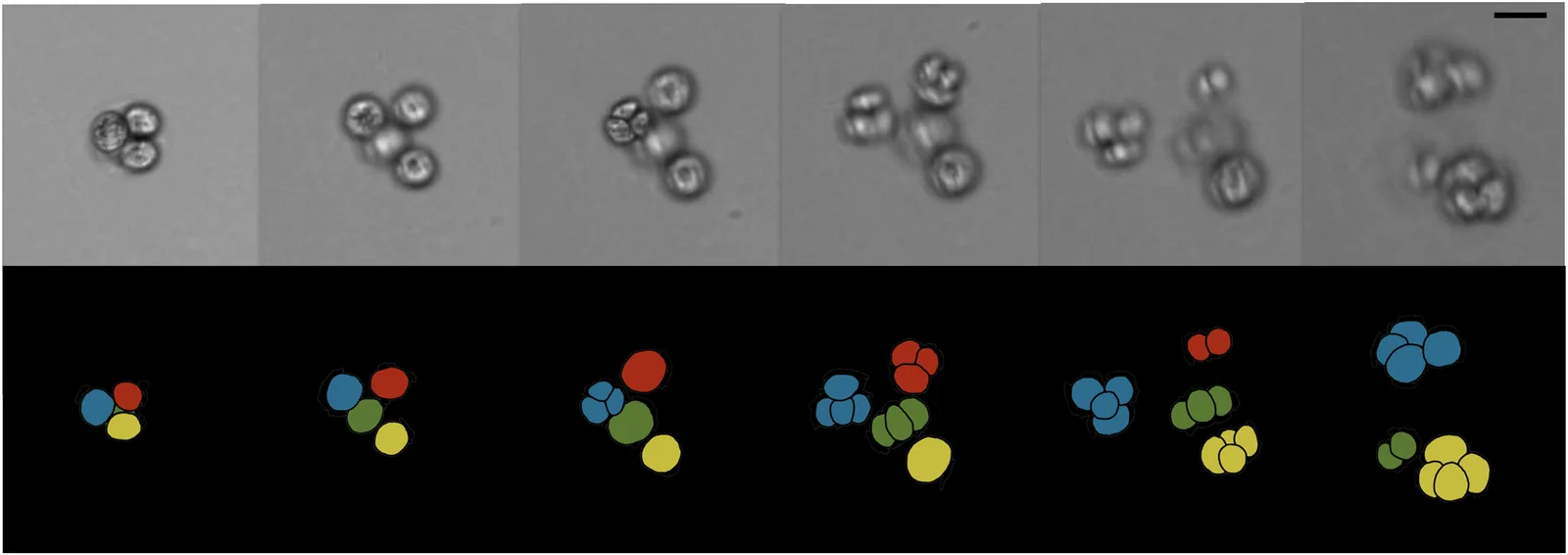
Evolved multicellular *C. reinhardtii* possess a multicellular life cycle The individual shown is a descendant from the 4× section of the Macroplate grown for over 70 generations in 1× viscosity. This demonstrates the heritable nature of the multicellular morphology. The top row shows stop-action stills of a video capturing the asexual reproduction of a single colony. Bottom row, graphical illustration of the same colony, with colors highlighting the daughter cell lineages derived from the cells within the mother colony. Scale bars equal 10 μm. Stills are derived from Video S2.

**Figure 6:**
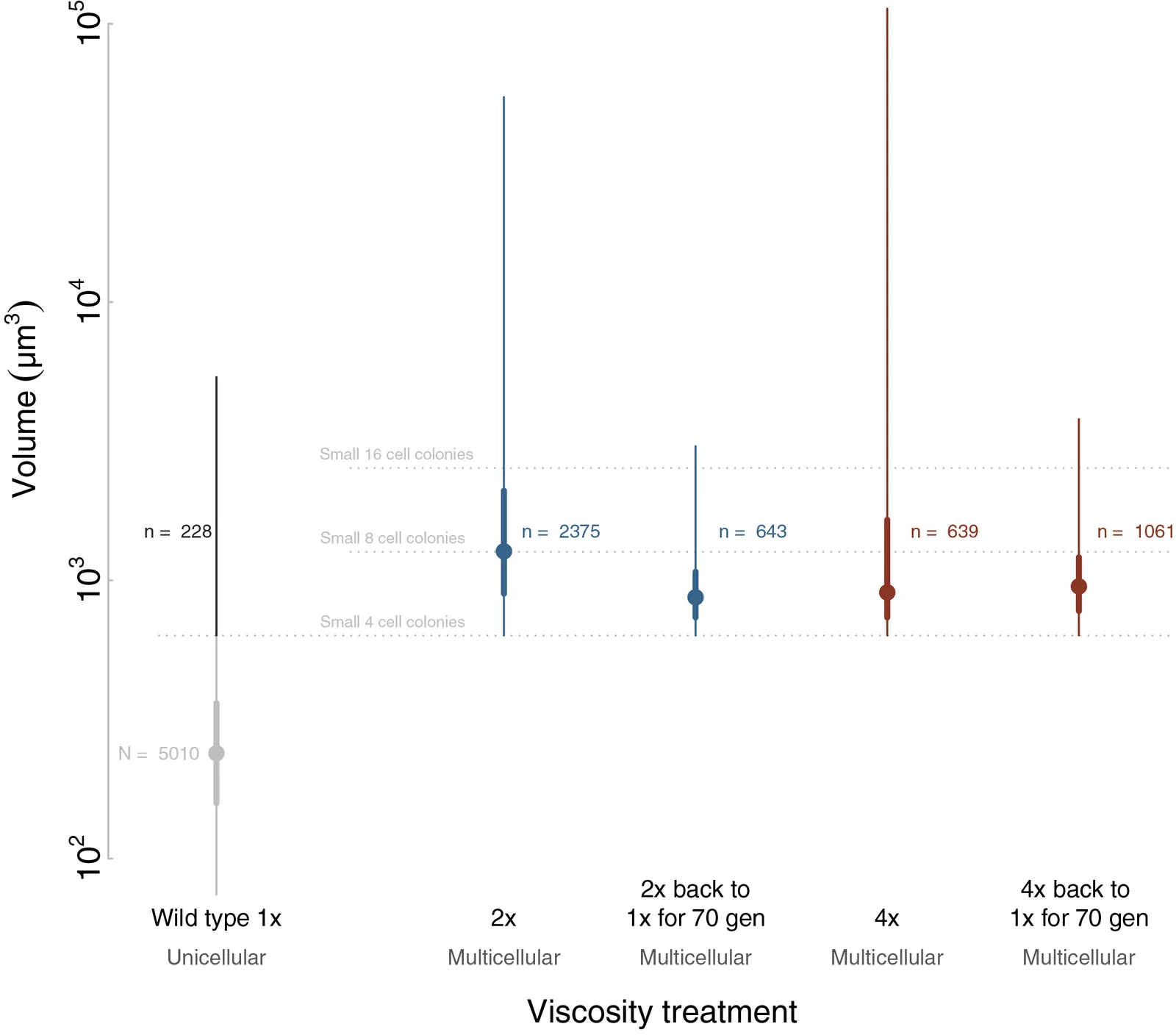
Heritability of motile multicellularity Average size of the wildtype populations of *C. reinhardtii* (“Wildtype 1×”) is shown on the left. The individuals that fall above the lower bound for the volume of a group of four cells are shown for both the wildtype population (*n* = 228) as well as the evolved populations from the Macroplate (“2×”: *n* = 2375, and “4×”: *n* = 639). Average size of the evolved populations of *C. reinhardtii* returned to media of 1× relative viscosity and transferred for over 70 generations is also shown (“2× back to 1× for 70 gen”: *n* = 643, and “4× back to 1× for 70 gen”: *n* = 1061). These populations show the heritability of the multicellular phenotype, as these motile multicellular phenotypes continued to be abundant in low viscosity.

Video S2. Time-lapse of evolved multicellular *C. reinhardtii* lifecycle

### Evolved multicellular organisms exhibit collective motility

In addition to unique size distributions, collective motility was observed in four- and eight-celled individuals from both 2× and 4× relative viscosities (Figure 7). As expected,^35^^,^^85^ single cells significantly (ANOVA: F _2,63_ = 5.869, *p* value = 0.0046; Tukey HSD: p-adj _2–1_ = 0.9353, p-adj _4–1_ = 0.0039, p-adj _4–2_ = 0.0285) decreased in speed as viscosity increased (∼10.8 μm s^−1^ in 1×, ∼4.6 μm s^−1^ in 2×, and ∼1.8 μm s^−1^ in 4× relative viscosities) whereas colonies of four and eight cells show no significant differences between viscosities (ANOVA: F _2,64_ = 3.351, *p* value = 0.0413; Tukey HSD: p-adj _2–1_ = 0.2525, p-adj _4–1_ = 0.0788, p-adj _4–2_ = 0.9837), maintaining average speeds near ∼15 μm s^−1^ in 2× and 4× relative viscosities (Video S1). The *p* value from the ANOVA being slightly less than 0.05 is a result of the multicellular speeds in both the 2× and 4× viscosities being slightly higher than the average speed of single cells in 1× viscosity, despite all average speeds being relatively similar. This is notable as motile multicellular colonies of *C. reinhardtii* are rarely—if ever—observed in natural or experimentally evolved populations.^95^

**Figure 7:**
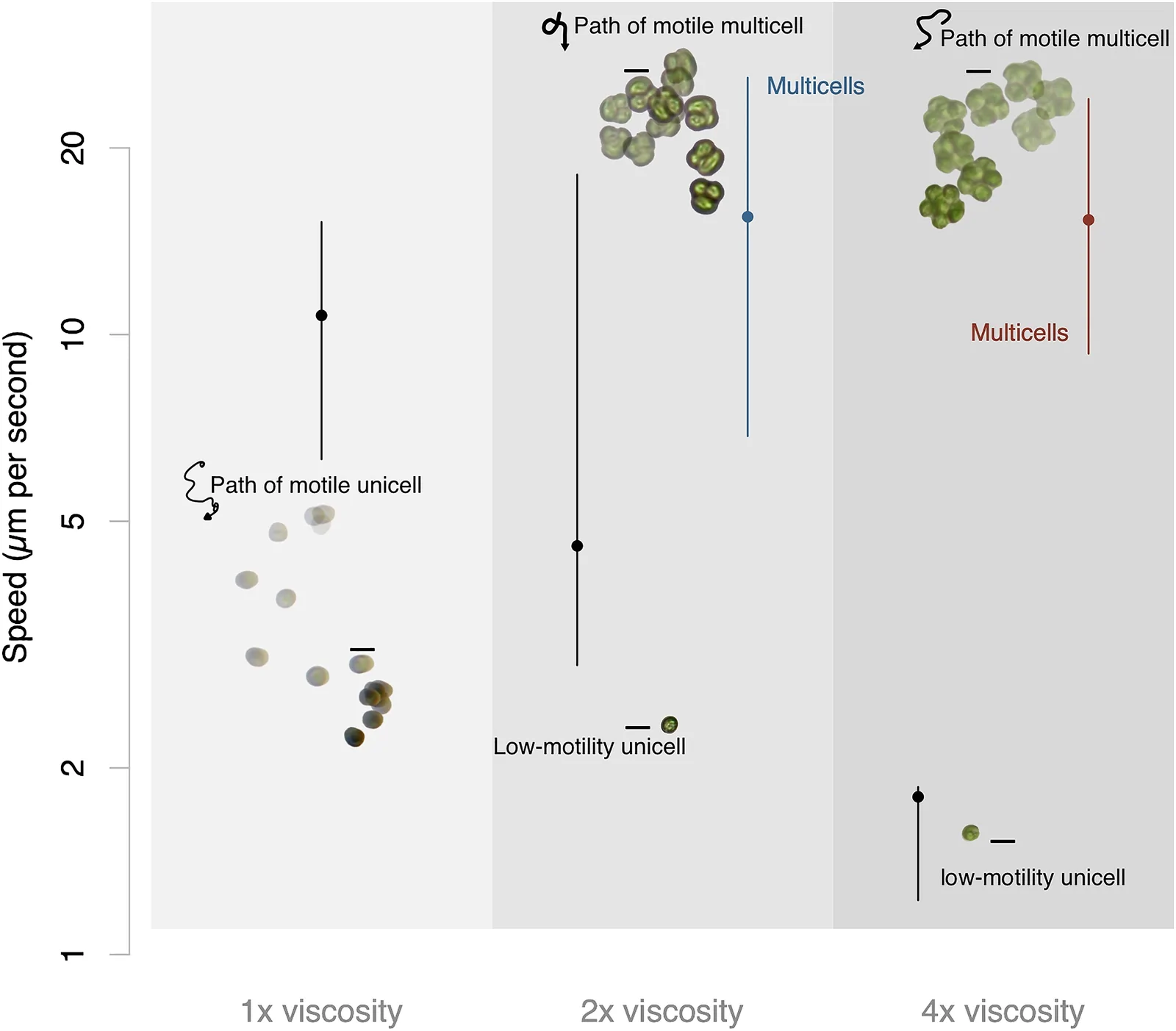
Quantification and visualization of motility in evolved multicellular populations of *C. reinhardtii* Microscopy videos analyzed by custom cell tracking code were used to find speeds of evolved individuals. Black data points show the decreasing average speeds of single cells from the Macroplate in their corresponding increasing viscosities (1× viscosity: *n* = 52 unicells tracked for 8 s each; 2× viscosity: *n* = 6 unicells tracked for 8 s each; 4× viscosity: *n* = 8 unicells tracked for 8 s each). Blue shows the average speed of motile multicells in 2× relative viscosity (*n* = 4 multicells tracked for 8 s each) and red shows the average speed of motile multicells in 4× relative viscosity (*n* = 11 multicells tracked for 8 s each). Motile unicellular phenotypes were the dominant morphology in the 1× viscosity section of the Macroplate. When viscosity increased to 2× relative viscosity, motile multicells of four emerged. In the 4× relative viscosity section of the Macroplate, motile multicells of both four and eight cells were abundant in the population. To highlight the motility we observe in multicellular colonies, we show example colonies as a stop-frame animation with about 10 frames between each image, and all phenotypes are displayed on the same scale (scale bars equal 10 μm). Single cells significantly (ANOVA: F _2,63_ = 5.869, *p* value = 0.0046; Tukey HSD: p-adj _2–1_ = 0.9353, p-adj _4–1_ = 0.0039, p-adj _4–2_ = 0.0285) decreased in speed as viscosity increased (∼10.8 μm s^−1^ in 1×, ∼4.6 μm s^−1^ in 2×, and ∼1.8 μm s^−1^ in 4× relative viscosities).

Video S1. Evolved motile multicellular *C. reinhardtii*

## Discussion

The results of our experiments clearly match or exceed two theoretical expectations. The first is that viscosity has a disproportionate effect on motility in single cells compared with multicells due to the increase in collective thrust.^91^ As viscosity increases, we observe single cells decrease in speed whereas multicells do not (Figure 7). The second expectation relates to the balance between nutrient acquisition and metabolism. The observed similarity in speed between multicells in high viscosity and unicells in low viscosity matches the theoretical prediction^35^ for where nutrient balance occurs. While metabolic work would be needed to empirically confirm nutrient balance, our results demonstrate the mechanism by which nutrient balance can be achieved—through the increased motility afforded by the collective action of multiple flagellated cells. Not only do we observe a disproportionate effect of viscosity on single cells versus multicells, but we also observe that multicells maintain (or exceed) a speed in high viscosity that predictably corresponds to the speed of single cells in low viscosity (Figure 7).

As discussed above, the fossil record of multicellular eukaryotes differs between algal clades and animals. Algal macrofossils extend well before the Cryogenian, but both their morphological disparity and diversity remained low until that time^28^^,^^34^ (Figure 1). With few exceptions (such as Bangiomorpha and Protocladus) it is difficult to distinguish between coenocytic, multinucleate, or truly multicellular forms.^44^^,^^45^ Because red algae lack flagella, whereas green algae ancestrally possessed them, we suspect that the selection for increased size observed in our experiment (achievable only through multicellularity) contributed to the radiation and rise in morphological disparity seen in green algal clades during the Cryogenian in line with divergence times of multicellular green algae derived from molecular clocks.^26^ The mechanism we identify could apply equally to sessile forms moved by water and to motile forms actively moving through it. Despite our use of *Chlamydomonas*, these results also shed light on the origins of animals, given the flagellated ancestry shared by early metazoans.

With the fossil record, we are able to observe “complexity” arise repeatedly throughout Earth’s history (Bangiomorpha, Proterocladus, Oingshania, and so forth). Our current definition of complexity can be molded to encompass a wide range of organisms, including life in both prokaryotic and eukaryotic clades. Our experiment couples the phenotypic aspects of multicellularity with a relevant environmental pressure that would not only result in multicellular phenotypes, but also provide an advantage to being complex. Snowball Earth provides a unique environmental filter that would favor motile complex eukaryotic life and provide an opportunity for ecological success. Lineages that go on to become complex typically demonstrate sustained increases in size and morphological complexity, as well as the potential to actively modify their local environment, creating novel ecological niches. These features are hallmarks of adaptive radiations^96^ and characteristics that likewise define the multicellular radiations of animals, plants, and macroalgae.

While we focus on the Cryogenian context, we are not claiming a direct evolutionary link between Snowball Earth and multicellularity in the volvocine algae specifically, which evolved in the Triassic.^97^ Instead, we use the experiment to assess whether viscosity-related selection could provide insights into the broader evolution of multicellularity across diverse eukaryotic lineages that share a flagellated bauplan with *Chlamydomonas*. This mechanism may not be universal, but likely depends on eukaryotic species possessing a restricted set of a broad parameter space defined by the scaling between speed and viscosity, body size and metabolic rate, and metabolic rate and temperature.^35^^,^^85^ For example, if the scaling between metabolic rate and temperature is strong, then there may be little evolutionary advantage to multicellularity for those organisms even under Snowball Earth conditions.^85^ It is not known how these scaling parameters vary across eukaryotic life, nor is it known how evolvable they are, but given the phylogenetic rarity of multicellularity in eukaryotes (six or so Neoproterozoic origins among many hundreds of clades extant at that time), it is likely that the specific combination of parameters that promote multicellular survival under cold, high-viscosity conditions is relatively rare.

The high viscosity of Snowball Earth oceans provided a universal selective barrier that could have been overcome using a variety of strategies. As both increased speed and decreased size are possible adaptations for high viscosity, it is not surprising to see these phenotypes (such as fast-moving ciliates^98^ and unusually small alpha cyanobacteria^99^) arise around this same time in Earth history. Other adaptive strategies for motility and nutrient acquisition likely evolved during this time as well.^75^ Many marine macroalgae rely on the surrounding current to bring nutrients to their blades^100^; high seawater viscosity may explain the increase in surface area to volume ratio observed in Cryogenian seaweeds.^28^

Traits we observe to evolve in response to high viscosity could have also been advantageous in the warm, post-glacial oceans. As glaciers melted at the end of the Cryogenian period, nutrients were rapidly released into coastal waters.^101^ The resulting eutrophic environments would have led to a burst in primary productivity.^62^ In this competitive environment, the novelty of being larger and moving faster in order to survive the cold, viscous conditions of Snowball Earth oceans could have led to an ecological innovation in the post-Snowball world. Nascent motile multicellular lineages could now exploit the lower viscosities of these waters to actively modify their immediate surroundings, potentially altering local fluid flow, concentrating resources, or repositioning themselves in light and nutrient gradients. This ability to better manipulate their surroundings^35^ would allow for feeding in new and previously inaccessible ways. The capacity to have more control over the local environment, rather than passively experience it, may have opened the door for the diversification and specialization of the dominant multicellular lineages on Earth today.

### Limitations of the study

Several limitations should be considered when interpreting our findings. First, our study focused on a modern, photosynthetic organism from a lineage with multicellular relatives. As a result, the extent to which these findings generalize across taxa, particularly animals and early multicellular lineages, remains to be determined. We also assessed morphological rather than genetic heritability, providing insight into the transmission of phenotypic variation without directly addressing its genetic basis. Lastly, our sampling captured a subset of the broader population, providing an important but necessarily limited perspective on population-level variation.

## Resource availability

### Lead contact

Further information and requests should be directed to and will be fulfilled by the Lead Contact, Andrea Halling (andrea.halling@utah.edu).

### Materials availability

This study did not generate new unique reagents.

### Data and code availability

All datasets have been deposited at Zenodo and are publicly available as of the date of publication at https://doi.org/10.5281/zenodo.19499855. Code available upon request.

## Acknowledgments

The authors would like to sincerly thank Will Ratcliff, Paul Hoffman, and three other anonymous reviewers for their extremely helpful and insightful feedback. We would also like to thank Adam Younkin for help fabricating the macroplate, Anton P. Avramov, Jeffrey Cameron, Joe Dragavon, and Jian Wei Tay for help with microscopy, Sarah Hurley, Sarah Leventhal, Jinchung Li, Pat Kocielek, and Rebecca Safran for helpful discussion and feedback. This work was funded by an 10.13039/100000001NSF GRFP to AH, and a WM Keck Foundation Grant to CS.

## Author contributions

Conceptualization and methodology: B.W., C.S., and A.H.

Investigation: A.H., B.G., and A.H.

Formal analysis: A.H., B.W., and C.S.

Writing and editing: all authors

## Declaration of interests

The authors declare no competing interests.

## STAR★Methods

### Key resources table

**Table:** 

REAGENT or RESOURCE	SOURCE	IDENTIFIER
**Experimental models: organisms/strains**		

*Chlamydomonas reinhardtii* (CC-125 mt+)	Chlamydomonas Resource Center	CC-125

**Chemicals, peptides, and recombinant protiens**		

Ficoll 400	Sigma-Aldrich	CAS number: 26873-85-8

**Other**		

HSA medium	Prepared in lab	https://www.chlamycollection.org/methods/media-recipes/hs-hsa-and-ya/
Beckman Coulter Multisizer 4s	Beckman Coulter	Multisizer 4s
Light microscope	Standard laboratory equipment	N/A

### Experimental model and study participant details

#### Experimental model organism

Wild-type *Chlamydomonas reinhardtii* (strain CC-125 mt+) was used in this study. The cell line used in this study was not independently authenticated or tested for mycoplasma contamination. However, we directly obtained our culture from the Chlamydomonas Resource Center which maintains authenticated stocks and high quality control practices. Cultures were grown in 10 mL of liquid HSA medium to an optical density (OD750) of approximately 0.7 (∼2 × 10^6^ cells/mL). Cultures were maintained under 500 nm light on a shaking platform at 100 rpm to promote motility. Culture life cycles were not synchronized to ensure a diversity of morphologies at the time of inoculation.

Individual cells are approximately 10 μm in diameter (volume ∼270 μm^3^), with observed motility speeds ranging from 10 to 40 μm s−1. Four-celled colonies are occasionally observed during division; however, due to flagellar constraints and the presence of the mother cell wall, outward-facing flagella enabling motility are rare. Information on the sex of this strain is not available and could not be determined, representing a limitation for generalizability.

No other experimental models/study participants (animals, human participants, plants, microbe strains, cell lines, primary cell cultures) were used in this study.

### Method details

#### Evolution experiment

A large circular petri plate (24.5 cm diameter) was constructed with radial step gradients of increasing viscosity (3.5 cm ring width). Ficoll 400 was added to 1.5% agar HSA medium to create a solid base layer with defined viscosities. This base layer was overlaid with a 3 mm liquid HSA swim layer.

The system was allowed to equilibrate for three days to permit diffusion of Ficoll 400 between layers, producing stable viscosity regions of approximately 1× (0.95 cP), 2× (2.3 cP), and 4× (4.1 cP) relative to water at 25°C. Plates were maintained under 500 nm illumination.

A 100 μL inoculum of *C. reinhardtii* culture was placed at the center of the plate. Populations expanded radially over 30 days (∼70 generations; generation time ∼10.5 h). Only individuals capable of propagating into higher viscosity regions continued to expand.

Population structure and phenotypes were assessed using Coulter Counter measurements, light microscopy, and MATLAB-based motion tracking.

#### Diffusion of Ficoll 400

Diffusion of Ficoll 400 from the agar layer into the swim layer was modeled using Fick’s second law of diffusion:

$$\varphi \left(X,t\right)=\varphi_{0}\;\mathrm{erf}\left(X/\left(2\sqrt{\left(Dt\right)}\right)\right)$$

Using D = 1.8 × 10^−7^ cm^2^/s and a diffusion distance of 1.5 mm, the time required to reach 0.1% of φ_0_ was calculated to be approximately 56.9 h (∼2.4 days). Plates were therefore equilibrated for three days prior to inoculation.

#### Phenotypic plasticity experiments

To isolate plastic responses, wild-type *C. reinhardtii* cultures were directly inoculated into media of 1×, 2×, and 4× viscosities. Nine 15 mm petri dishes (three per treatment) were prepared under identical light conditions. Cultures were grown for ∼7 generations before sampling for Coulter Counter and microscopy analyses.

#### Media preparation

HSA medium was used throughout. Ficoll 400 was added to agar base layers at 0% (1×), 5.5% (2×), and 9% (4×). A uniform 3 mm swim layer of HSA medium was added above the base layer. For plasticity experiments, Ficoll 400 was added directly to liquid media at the same concentrations.

Viscosities were measured as 0.95 cP (1×), 2.3 cP (2×), and 4.1 cP (4×).

#### Post-experiment transfers

Samples from 1×, 2×, and 4× regions were transferred into new petri dishes and 10 mL culture tubes containing corresponding viscosities. Additional samples were transferred into 1× viscosity media and maintained for ∼70 generations to assess phenotypic stability. Size distributions after transfer were compared to wild-type controls to evaluate heritability.

#### Coulter counter measurements

Size distributions were measured using a Beckman Coulter Multisizer 4s. Samples were analyzed using 200 μm, 1000 μm, and 2000 μm aperture tubes to capture a broad size range.

For the 200 μm aperture, equal numbers of cells were counted across treatments. For larger apertures, fixed volumes were analyzed and individuals grouped into size classes. Sampling volumes were 1 mL total with appropriate electrolyte solutions.

#### Microscopy and motility analysis

Motility was quantified using light microscopy and MATLAB-based tracking. For each viscosity treatment, five predefined fields of view were recorded for 10 s each. Videos were processed to remove background noise, and tracking algorithms were used to quantify cell size and velocity. Cell counts within multicellular clusters were manually verified.

### Quantification and statistical analysis

Statistical analyses are reported in the results section and figure legends. These include chi-squared tests, Kolmogorov-Smirnov tests, Fisher’s exact tests, and ANOVA with Tukey HSD post hoc comparisons.

Sample sizes (n), definitions of n (e.g., number of cells, colonies, or measurements), and measures of dispersion are provided in the Results and corresponding figures. Significance thresholds and exact *p*-values are reported where applicable.

No explicit randomization or blinding procedures were implemented. Inclusion criteria consisted of all measurable individuals within sampled populations; no data were excluded unless corrupted by imaging or measurement artifacts.

### Additional resources

No additional external resources were generated for this study.

## Supplemental information

Table S1. Raw data of Coulter Counter size distributions from evolved and plastic populations

Table S2. Raw data of Coulter Counter size distributions from evolved populations placed back into low, 1× viscosity for 70 generations

Table S3. Raw data of speeds calculated from microscopy videos
